# Screening and Diagnostic Mammography During Pregnancy and Lactation: A Systematic Review of the Literature

**DOI:** 10.7759/cureus.66465

**Published:** 2024-08-08

**Authors:** Menelaos Zafrakas, Panayiota Papasozomenou, Angeliki Gerede, Themistoklis Mikos, Apostolos Athanasiadis, Grigoris Grimbizis

**Affiliations:** 1 1st Department of Obstetrics and Gynecology, Aristotle University of Thessaloniki, Thessaloniki, GRC; 2 Obstetrics and Gynecology, School of Health Sciences, International Hellenic University, Thessaloniki, GRC; 3 3rd Department of Obstetrics and Gynecology, Aristotle University of Thessaloniki, Thessaloniki, GRC; 4 Obstetrics and Gynecology, Aristotle University of Thessaloniki, Thessaloniki, GRC; 5 1st Department of Obstetrics and Gynecology, Medical School, Aristotle University of Thessaloniki, Thessaloniki, GRC

**Keywords:** breast cancer, lactation, pregnancy, breast cancer screening, mammography

## Abstract

In recent years, the age of childbearing has been increasing in Western countries, and consequently the need to conduct mammography during pregnancy and lactation is also increasing. The aim of the present study was to systematically review the existing evidence regarding the overall use of mammography during pregnancy and lactation. A systematic review of the literature was conducted in PubMed, Epistemonikos, and clinicaltrials.gov, by using the search terms “pregnancy” AND “mammography”, and “lactation” AND “mammography”. The review protocol was prospectively registered in PROSPERO (CRD42024543971). Initially, 1,038 articles were identified; the titles and abstracts of 441 studies were screened; 40 studies were retrieved; after assessment of full texts, 20 studies were included for data extraction and further analysis. All 20 studies were retrospective; 14 studies included women with pregnancy-associated breast cancer, five studies included women with breast symptoms during pregnancy and/or lactation and one study included young breast cancer patients under age 40. Overall, 420 diagnostic and one incidental screening mammography examinations were performed during pregnancy and/or lactation with a 78.6% cumulative detection rate of breast cancer. The role of mammography was confounded by the use of breast ultrasound in most studies. In conclusion, the use of mammography during pregnancy and lactation is based on empirical data from retrospective studies, not directly addressing this issue. Hence, well-designed, focused, prospective clinical studies are needed in order to improve existing evidence regarding the use of diagnostic and screening mammography during pregnancy and lactation.

## Introduction and background

Breast cancer is the most commonly diagnosed malignancy and the leading cause of cancer deaths in women worldwide; it ranks first in 157 countries for incidence and first in 112 countries for mortality [1]. Mammography is the most important method for breast cancer screening, as it is the only modality with demonstrated mortality reduction, based on data from randomized clinical trials [2-4]. Most authorities recommend initiation of screening mammography for average-risk women at age 40 [5, 6], and even earlier for high-risk women with a family history of breast cancer, taking into account that 8.4% of breast cancer cases occur in women aged 35-44 years old and that breast cancer incidence is increasing with increasing age [7]. Besides screening, mammography is widely used in the diagnostic workup of women with signs and symptoms of benign breast disease and/or breast cancer.

In recent years, the fertility rate of women aged 35-39 and ≥40 years is increasing both in the European Union [8] and the USA [9]. It is anticipated that this trend will continue in the future, as a consequence of the increasing use of oocyte donation and oocyte cryopreservation, commonly referred to as “social freezing”, so that even more women will be pregnant or lactating in their 40s [10-12]. Furthermore, trends in rates and duration of breastfeeding have been steadily increasing in recent years [13].

The American College of Radiology Expert Panel on Breast Imaging considers mammography to be safe during pregnancy and recommends that breast cancer screening should be tailored to patient age and breast cancer risk [14]. More recently, the United States National Comprehensive Cancer Network (NCCN) published its updated guidelines on breast cancer screening and included the recommendation of providing mammography to women during pregnancy and lactation [15]. However, evidence-based data from clinical trials investigating the use of screening and diagnostic mammography during pregnancy and lactation appear to be scarce. In particular, the German Working Group for Gynecological Oncology gives a Grade C recommendation to the statement “Breast imaging and biopsy like as in non-pregnant patients”, with Level of Evidence (LoE) category 4, without explicitly mentioning mammography [16]. Given the ever-increasing number of women in Europe and North America getting pregnant and breastfeeding in their 40s and late 30s and the paucity of evidence-based data on this issue, the purpose of the present study was to carry out a systematic review of clinical studies regarding the use of mammography in pregnancy and lactation.

## Review

Materials and methods

The present systematic review of the literature has been prospectively registered in the Prospective Register of Systematic Reviews (PROSPERO) (CRD42024543971) and it is reported according to the Preferred Reporting Items for Systematic Reviews and Meta-Analyses (PRISMA) statement [17]. The review question was the following: What is the body of clinical evidence supporting the use of mammography in pregnant and lactating women? The following databases were searched: PubMed, Epistemonikos, and clinicaltrials.gov. The following search terms were used: 1) “pregnancy” AND “mammography”, and 2) “lactation” AND “mammography”. Identified studies were then filtered in order to include only papers in English, studies in humans, and studies in females 19-44 years old. There was no restriction regarding publication dates. Retrieved studies and relevant review articles were hand searched for further studies. The following inclusion criteria were set: randomized controlled trials, observational studies, case control studies and cohort studies. The following exclusion criteria were used: Clinical Guidelines, any type of review articles, case reports and case series. In addition, PubMed, the Cochrane Library and PROSPERO were searched for any published review articles focusing on the use of mammography in pregnancy and lactation, by using the search term “mammography” for review articles and then screening the titles for the use of mammography in pregnancy and lactation.

The PICO criteria, i.e. Participants/Population, Intervention(s), Comparator(s)/control, and Outcomes were used for the selection of studies, as follows. Participants/population: Pregnant and lactating women undergoing screening or diagnostic mammography; Intervention(s): Screening or diagnostic mammography; Comparator(s) / control: Pregnant and lactating women who did not undergo screening or diagnostic mammography; Outcome(s): Diagnosis of breast cancer during pregnancy and lactation.

The titles and abstracts of studies identified by using the search strategy described above were screened independently by two authors (MZ and PP) in order to identify studies that meet the inclusion criteria, and conflicts were resolved by discussion or referral to a third author (AG). The full texts of identified studies were retrieved and independently assessed for eligibility by two authors (MZ and PP), and conflicts were resolved by discussion or referral to a third author (AG), and justification of exclusion was documented.

Risk of bias (quality) assessment: Two reviewers (MZ and PP) independently assessed the risk of bias using the Risk Of Bias In Non-randomised Studies of Interventions (ROBINS-1) tool in included studies [18]. Disagreements between the two authors (MZ and PP) over the risk of bias in particular studies were resolved by discussion or referral to a third reviewer (AG).

Data from studies were extracted independently by two authors (MZ and PP) and conflicts were resolved by discussion or referral to a third author (AG). Data extracted included first author, year of publication, country where the study was conducted, study design, total number of breast cancer patients, number of patients with breast cancer diagnosed during pregnancy, number of patients with breast cancer diagnosed during lactation and/or postpartum, age of patients, number of diagnostic and number of screening mammography examinations performed during pregnancy and/or lactation, sensitivity and specificity of mammography for breast cancer, and if any other imaging studies were used.

Results

**Figure 1:**
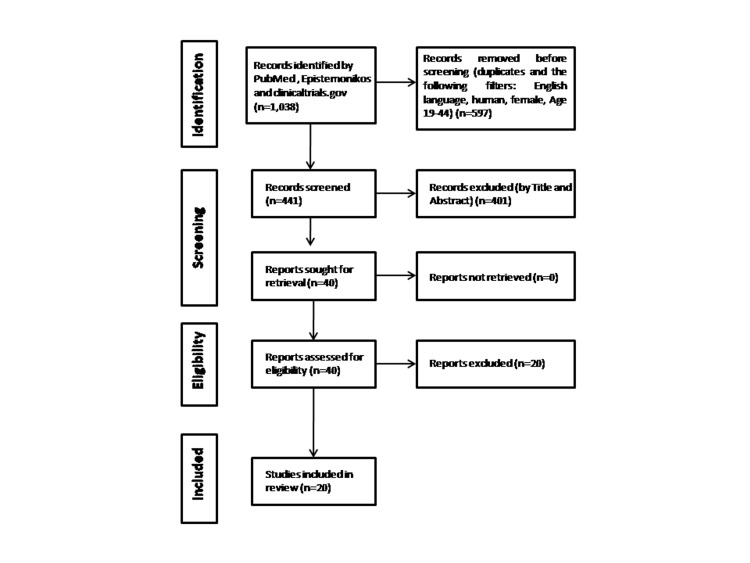
PRISMA flowchart presenting the study search, screening and selection process

The study search, screening and selection process is presented in the PRISMA flowchart in Figure 1.

In total, 1,038 articles were identified using the following keywords in PubMed, Epistemonikos, and clinicaltrials.gov: “pregnancy” AND “mammography”, and “lactation” AND “mammography”. After removing duplicate entries, articles not written in English, studies not in human subjects, and articles with participants’ age other than 18-44 years (n=597), 441 studies were left for screening. After screening the titles and abstracts, 401 studies were excluded and 40 studies were sought for retrieval; the list of these 40 studies is presented in Appendix 1. All 40 studies were retrieved and after assessment of the full text, 20 studies were excluded and 20 studies were selected for final analysis according to the study inclusion and exclusion criteria. The reasons for the exclusion of retrieved studies are presented in Appendix 2. In brief, six studies were excluded because they were case reports or case studies; three studies because they were reviews of the literature; and 11 due to study content. No additional studies were found after searching the literature of retrieved studies and relevant reviews. It is noteworthy that there were no relevant randomized controlled trials identified in clinicaltrials.gov and PubMed by choosing the relevant filter. Furthermore, there were no published review articles focusing on the use of mammography in pregnancy and lactation found in PubMed, the Cochrane Library, and PROSPERO.

An overview of the risk of bias quality assessment of the 20 included studies is presented in Table 1. As mentioned above, since there were no randomized controlled trials identified, the Risk Of Bias In Non-randomised Studies of Interventions (ROBINS-1) tool [18] was used to assess all 20 included studies. Details on the risk of bias quality assessment of the included studies according to the ROBINS-1 tool [18] are presented in Appendices 3 and 4. In brief, two studies had critical, 14 serious, and four moderate risk of bias.

**Table 1:** Overview of the risk of bias quality assessment of the 20 included studies according to the ROBINS-1 tool SR = Serious risk, CR = Critical risk, MR = Moderate Risk, L = Low Risk

Study	Domain 1 confounding	Domain 2 selection of participants	Domain 3 classification of interventions	Domain 4 deviations from intended interventions	Domain 5 missing data	Domain 6 measurement of outcomes	Domain 7 selection of reported result	Overall
Hu et al. 2021 [19]	CR	CR	SR	MR	CR	CR	SR	SR
Chung et al. 2020 [20]	CR	SR	LR	LR	MR	MR	MR	MR
Reyes et al. 2020 [21]	CR	CR	SR	SR	MR	MR	SR	SR
Taşkın et al. 2019 [22]	CR	CR	SR	SR	MR	MR	SR	SR
Wang et al. 2019 [23]	CR	CR	SR	SR	MR	MR	SR	SR
Johansson et al. 2019 [24]	CR	CR	SR	SR	CR	CR	SR	CR
Pugh et al. 2018 [25]	CR	CR	SR	SR	CR	CR	SR	CR
Myers et al. 2017 [26]	CR	CR	LR	LR	MR	MR	MR	MR
Langer et al. 2014 [27]	CR	CR	SR	SR	MR	MR	SR	SR
Córdoba et al. 2013 [28]	CR	CR	SR	SR	MR	MR	SR	SR
Taylor et al. 2011 [29]	CR	CR	SR	SR	MR	MR	SR	SR
Robbins et al. 2011 [30]	CR	SR	LR	LR	MR	MR	MR	MR
Son et al. 2006 [31]	CR	CR	SR	SR	MR	MR	SR	SR
Yang et al. 2006 [32]	CR	CR	SR	SR	MR	MR	SR	SR
Bock et al. 2006 [33]	CR	SR	LR	LR	MR	MR	MR	MR
Obenauer & Dammert 2006 [34]	CR	CR	SR	SR	MR	MR	SR	SR
Ahn et al. 2003 [35]	CR	CR	SR	SR	MR	MR	SR	SR
Samuels et al. 1998 [36]	CR	CR	SR	SR	MR	MR	SR	SR
Liberman et al. 1994 [37]	CR	CR	SR	SR	MR	MR	SR	SR
Ishida et al. 1992 [38]	CR	CR	SR	SR	MR	MR	SR	SR

Data extracted from the 20 included studies are presented in Table 2. Regarding the year of publication eight studies were published after 2015 [19-26], eight studies were published between 2005 and 2014 [27-34] and four studies were published between 1992 and 2004 [35-38]. Regarding the country where studies were conducted, eight studies were carried out in North America (seven in the USA [19, 20, 25-26, 30, 32, 37] and one in Canada [36]), six studies in Europe (two in Spain [21, 28], two in Germany [33-34], one in Sweden [24], and one in France [27]), five in Asia (two in South Korea [31, 35], one in Turkey [22], one in China [23], and one in Japan [38]), and one study was carried out in Australia [29].

**Table 2:** Data extracted from the 20 included studies n.a. = not available; BUS = Breast Ultrasound; MRI = Magnetic Resonance Imaging ^*^PABC = Pregnancy-Associated Breast Cancer, defined as breast cancer during pregnancy and during the first year postpartum. ^ǂ^ 135 women diagnosed with breast cancer during pregnancy or within one year postpartum; in total there were 273 women diagnosed with breast cancer during pregnancy and during the first two years postpartum and 273 matched controls. ^¥^ There was a case of malignant breast lymphoma missed by mammography (characterized as BIRADS-3) ^§^ median

Study	Country	Study design	Number of breast cancer patients			Age mean (range)	Mammography exams				Other imaging
			Total	Pregnant	Lactating		Diagnostic	Screening	Sensitivity	Specificity	
Hu et al. 2021 [19]	USA	Retrospective (single center)	145	10	14	34.6 (<40)	24	0	n.a.	n.a.	BUS
Chung et al. 2020 [20]	USA	Retrospective (single center)	5 (out of 167)	0	5	35.1 (17-52)	98	0	100% (5/5)	61%	BUS
Reyes et al. 2020 [21]	Spain	Case Control (single center)	42	42	0	37 (n.a.)	36	0	56.5% (13/23)	n.a.	BUS
Taşkın et al. 2019 [22]	Turkey	Retrospective (single center)	47	9	38 PABC* postpartum	32 (25-44)	47	0	87.2% (41/47)	n.a.	BUS, MRI
Wang et al. 2019 [23]	China	Retrospective (single center)	142	30	112	30.3 (24-44)	48	0	83.3% (40/48)	n.a.	BUS
Johansson et al. 2019 [24]	Sweden	Case Control (Registry)	135ǂ	41	94 PABC* postpartum	n.a. (15-44)	105	0	n.a	n.a.	BUS
Pugh et al. 2018 [25]	USA	Retrospective (Registry)	65 PABC*	n.a.	n.a.	34.9 (n.a.)	4	0	n.a.	n.a.	No
Myers et al. 2017 [26]	USA	Retrospective (single center)	53	9	24	36 (29-43)	32	1	91% (30/33)	n.a.	BUS, MRI
Langer et al. 2014 [27]	France	Retrospective (single center)	113	21	80 PABC* postpartum	33.7 (24-42)	89	0	80.9% (72/89)	n.a.	BUS
Córdoba et al. 2013 [28]	Spain	Retrospective (single center)	25	25	0	36 (23-48)	24	0	66% (15/24)	n.a.	BUS
Taylor et al. 2011 [29]	Australia	Retrospective population study	22	10	6 (and 5 non-lactating)	35 (28-40)	19	1	74% (14/19)	n.a.	BUS, MRI
Robbins et al. 2011 [30]	USA	Retrospective (single center)	4 (out of 147)	1	2 (and 1 postpartum)	32.3 (19-47)	85	0	100% (4/4))	93%	BUS
Son et al. 2006 [31]	S. Korea	Retrospective (single center)	6 (out of 49)	6	0	31.4 (23-37)	5	0	20% (1/5)	n.a.	BUS
Yang et al. 2006 [32]	USA	Retrospective (single center)	23	23	0	34§ (24-45)	20	0	90% (18/20)	n.a.	BUS
Bock et al. 2006 [33]	Germany	Retrospective (single center)	5 (out of 25)	5	0	33.8 (30-38)	18	0	100% (5/5)¥	100%	BUS
Obenauer & Dammert 2006 [34]	Germany	Retrospective (single center)	2 (out of 27)	0	0	33 (25-41)	18	0	100% (2/2)	n.a.	BUS, MRI
Ahn et al. 2003 [35]	S. Korea	Retrospective (single center)	22	10	12	33 (26-49)	15	0	86.7% (13/15)	n.a.	BUS
Samuels et al. 1998 [36]	Canada	Retrospective (single center)	19	10	4 (1 n.a. & 4 postpartum)	31 (23-41)	8	0	62.5% (5/8)	n.a.	BUS
Liberman et al. 1994 [37]	USA	Retrospective (single center)	85	12	19	34 (24-41)	23	0	78% (18/23)	n.a.	BUS
Ishida et al. 1992 [38]	Japan	Case Control (multi-center)	192	72	120	32.3 (n.a.)	50	0	68% (34/50)	n.a.	BUS
Total			1152	330	541		764	1	78.6% (330/420)		

Regarding study design, 17 were retrospective cohort studies (15 were conducted in a single center [19-20, 22-23, 26-28, 30-37], one was registry-based [25], and one was population-based [29]) and three were case-control studies (one in a single center [21], one was registry-based [24], and one in multiple centers [38]). In 14 studies [21-29, 32, 35-38] the study population consisted of patients with pregnancy-associated breast cancer, i.e. breast cancer diagnosed during pregnancy and within one or two years after delivery, with the total number of patients ranging between 19 and 273; five studies [20, 30-31, 33-34] included women with breast symptoms during pregnancy and/or lactation, with the number of breast cancer cases ranging between two and six, and the total number of women presenting with breast symptoms ranging between 27 and 167; finally, in one study the study population consisted of 145 young breast cancer patients under age 40, with 10 cases diagnosed during pregnancy and 14 during lactation. The mean patient age across studies was between 30.3 and 37 years.

Overall, the number of diagnostic mammography examinations performed during pregnancy and/or lactation ranged between 5 and 98, while there was only one incidental case of mammography for breast cancer screening performed during lactation [26]. The detection rate of breast cancer with mammography varied between 20 and 100%; the cumulative detection rate of breast cancer with mammography performed during pregnancy and lactation was 78.6% (330 out of 420 breast cancer cases). In two case-control studies, the sensitivity of mammography was lower in pregnant breast cancer patients as compared with non-pregnant patients in the control groups; 68% vs. 74% [38] and 56.5% vs. 61.7% [21]. Specificity of mammography was reported only in three studies and it was 61% [20], 93% [30] and 100% [33]. Breast ultrasound was also performed in 19 studies [19-24, 26-38], breast MRI in four studies [22, 26, 29, 34], while there was only one study in which mammography was the only imaging method used [25]. In all four studies [22, 26, 29, 34], MRI was performed postpartum, except one case in one study [29] where MRI was performed during the first trimester of a pregnancy that was later terminated.

Discussion

Breast cancer is the most common malignancy diagnosed during pregnancy [39-40]. The term "pregnancy-associated breast cancer" (PABC) has been used for years and includes those cases diagnosed during pregnancy as well as those diagnosed within one [27] or two [24] years after delivery, irrespective of whether a woman is breastfeeding or not. However, the use of this term has been challenged recently, and investigators with expertise in the field proposed that the term PABC should no longer be used [41]. Given that women in Europe and North America are increasingly postponing pregnancy to a more advanced age [9-10] and that most authorities recommend starting breast cancer screening at the age of 40 [6-7, 42], the possibility that mammography may have to be used during pregnancy and lactation is also increasing.

In the present systematic review, data from 20 relevant primary studies were extracted and analyzed further [19-38]. By using a comprehensive search strategy, we did not identify any randomized clinical trials or any other prospective study evaluating the role of screening and diagnostic mammography during pregnancy and lactation. Furthermore, all 20 studies included in the present systematic review addressed the review question rather indirectly. Thus, it was not surprising that the quality assessment of the included studies raised serious concerns of potential bias.

In five studies included in the present systematic review, mammography was performed in women with breast symptoms during pregnancy and/or lactation [20, 30-31, 33-34], but the total number of women with breast cancer in these studies was rather low, ranging between two and six. In 14 other studies [21-29, 32, 35-38], mammography was performed in women with breast cancer diagnosed during pregnancy and/or lactation and/or within one or two years after delivery, with the total number of patients ranging between 19 and 273; however, mammography was not performed in all these women, since most patients were examined with breast ultrasound with or without mammography [19-24, 25-38] and in four studies also with breast MRI postpartum [22, 26, 29, 34]. In total, mammography was performed in 420 pregnant and lactating women with breast symptoms yielding 330 breast cancer cases, with a cumulative detection rate of 78.6%; there was only one coincidental case of mammography performed for screening during lactation [26]. The specificity of mammography was reported only in three studies [20, 30, 33]. It is worth noting that in one of these studies [20], the addition of mammography to ultrasound in a cohort of lactating women lowered specificity from 67% to 61%. In contrast, the other two studies included both pregnant and lactating women and specificity was higher for mammography than ultrasound, i.e. 93% vs. 86% [30] and 100% vs. 89% [33]; this discrepancy may be due to differences in study populations and different equipment used. Taken together, these findings suggest that evidence regarding the role of diagnostic mammography in pregnancy and lactation is rather limited and blurred by the confounding effect of ultrasound. Moreover, there is in essence no direct evidence supporting the use of mammography for screening in this specific population of women.

Although breast ultrasound is considered to be the method of choice in evaluating breast symptoms during pregnancy and lactation [43], still mammography may provide additional diagnostic clues or even detect lesions not visible on breast ultrasound. Intriguingly, the latest NCCN guidelines for breast cancer screening recommend mammography but not ultrasound during pregnancy and lactation [16]. However, in the setting of breast cancer screening of the general population, the role of breast ultrasound has been evaluated in clinical trials mainly in combination with mammography, rather than as a standalone method, and it is firmly established that breast ultrasound detects additional cancers [44]. In pregnancy and lactation, neither mammography nor breast ultrasound have been evaluated for breast cancer screening. Ideally, a randomized controlled trial would be desirable for this purpose, but such a trial would need a large number of healthy pregnant and/or lactating women in their 40s to be recruited and many years of follow-up, in order to find out whether screening this population would lead to a reduction in breast cancer mortality with an acceptable false positive rate; a prospective cohort study in pregnant and lactating women would be a plausible alternative, yet even this would not be an easy task to be accomplished.

Due to certain practical technical issues, it remains to be seen if and to what extent mammography will be used in everyday practice for screening and diagnosis of breast cancer during pregnancy and lactation. It is well established that the scatter dose of radiation emitted during standard full-field digital mammography affecting the fetus is very low [45]. However, are pregnant women and their obstetricians alike willing to discuss the option of performing a mammography in the first trimester of pregnancy just for breast cancer screening or breast symptoms already clarified by the use of breast ultrasound? Regarding lactation, it has been advocated that the breasts should be emptied from milk before mammography [46]; this is dictated by common sense in order to avoid milk ejection during breast compression between the mammography plates, but does this practice influence in any way the diagnostic accuracy of mammography? Needless to say that no data exist regarding the use of novel, increasingly used mammographic techniques, such as digital breast tomosynthesis (DBT) and contrast-enhanced mammography (CEM) during pregnancy and lactation, especially since the use of contrast media during pregnancy is contraindicated [46].

The main limitations of the present systematic review were that the comprehensive search of the literature did not yield any studies directly addressing the review question, that all studies found were retrospective in design, including only two case-control studies reporting the sensitivity of mammography in pregnant breast cancer patients as compared with non-pregnant controls. On the other hand, the strength of this pre-registered systematic review is its originality, since to the best of our knowledge and after the appropriate search described above no such study has been published thus far.

## Conclusions

In the present systematic review evaluating the use of mammography in pregnancy and lactation only retrospective clinical studies, not directly addressing the review question were identified; in particular, only evidence regarding the role of diagnostic mammography in pregnancy and lactation was found, and its role was confounded by the in parallel use of breast ultrasound; furthermore, no substantial direct evidence regarding screening mammography in pregnancy and lactation was found. Hence, well-designed prospective clinical studies are needed in order to delineate the role of diagnostic and screening mammography during pregnancy and lactation.

## Appendices

Appendix 1: List of 40 studies sought for retrieval

1. Breast Imaging and Intervention during Pregnancy and Lactation. Peterson MS, Gegios AR, Elezaby MA, Salkowski LR, Woods RW, Narayan AK, Strigel RM, Roy M, Fowler AM. Radiographics. 2023 Oct;43(10):e230014. doi: 10.1148/rg.230014. PMID: 37708073

2. Application of nanotechnology in breast cancer screening under obstetrics and gynecology through the use of CNN and ANFIS. Zheng N, Yao Z, Tao S, Almadhor A, Alqahtani MS, Ghoniem RM, Zhao H, Li S. Environ Res. 2023 Oct 1;234:116414. doi: 10.1016/j.envres.2023.116414. Epub 2023 Jun 28. PMID: 37390953

3. Gestational breast cancer: current challenges in staging and treatment of breast cancer. Schad A, Slostad J, Rao R. BMJ Case Rep. 2020 Nov 3;13(11):e235308. doi: 10.1136/bcr-2020-235308. PMID: 33148569

4. Presentation and characteristics of breast cancer in young women under age 40. Hu X, Myers KS, Oluyemi ET, Philip M, Azizi A, Ambinder EB. Breast Cancer Res Treat. 2021 Feb;186(1):209-217. doi: 10.1007/s10549-020-06000-x. Epub 2020 Nov 2. PMID: 33136248

5. Breast cancer during pregnancy: matched study of diagnostic approach, tumor characteristics, and prognostic factors. Reyes E, Xercavins N, Saura C, Espinosa-Bravo M, Gil-Moreno A, Cordoba O. Tumori. 2020 Oct;106(5):378-387. doi: 10.1177/0300891620925158. Epub 2020 Jul 6. PMID: 32623975

6. Pregnancy-associated breast cancer: A review of 47 women. Taşkın F, Polat Y, Erdoğdu İH, Soyder A. Clin Imaging. 2019 Nov-Dec;58:182-186. doi: 10.1016/j.clinimag.2019.07.012. Epub 2019 Aug 5. PMID: 31404824

7. Clinicopathological characteristics, diagnosis, and prognosis of pregnancy-associated breast cancer. Wang B, Yang Y, Jiang Z, Zhao J, Mao Y, Liu J, Zhang J. Thorac Cancer. 2019 May;10(5):1060-1068. doi: 10.1111/1759-7714.13045. Epub 2019 Mar 28. PMID: 30920126

8. Misconceptions surrounding pregnancy-associated breast cancer. Khalil N, Fowler C. BMJ Case Rep. 2018 Dec 17;11(1):e226719. doi: 10.1136/bcr-2018-226719. PMID: 30567280

9. Diagnostic pathways and management in women with pregnancy-associated breast cancer (PABC): no evidence of treatment delays following a first healthcare contact. Johansson ALV, Weibull CE, Fredriksson I, Lambe M. Breast Cancer Res Treat. 2019 Apr;174(2):489-503. doi: 10.1007/s10549-018-05083-x. Epub 2018 Dec 14. PMID: 30552644

10. Characteristics and diagnosis of pregnancy and lactation associated breast cancer: Analysis of a self-reported regional registry. Pugh AM, Giannini CM, Pinney SM, Hanseman DJ, Shaughnessy EA, Lewis JD. Am J Surg. 2018 Oct;216(4):809-812. doi: 10.1016/j.amjsurg.2018.07.060. Epub 2018 Sep 20. PMID: 30270029

11. Background parenchymal enhancement in pregnancy-associated breast cancer: a hindrance to diagnosis? Taron J, Fleischer S, Preibsch H, Nikolaou K, Gruber I, Bahrs S. Eur Radiol. 2019 Mar;29(3):1187-1193. doi: 10.1007/s00330-018-5721-7. Epub 2018 Sep 18. PMID: 30229271

12. Breast cancer and pregnancy. Knabben L, Mueller MD. Horm Mol Biol Clin Investig. 2017 Aug 29;32(1):/j/hmbci.2017.32.issue-1/hmbci-2017-0026/hmbci-2017-0026.xml. doi: 10.1515/hmbci-2017-0026. PMID: 28850544

13. Imaging Appearance and Clinical Impact of Preoperative Breast MRI in Pregnancy-Associated Breast Cancer. Myers KS, Green LA, Lebron L, Morris EA. AJR Am J Roentgenol. 2017 Sep;209(3):W177-W183. doi: 10.2214/AJR.16.17124. Epub 2017 Jun 13. PMID: 28609163

14. JOURNAL CLUB: Scatter Radiation Dose From Digital Screening Mammography Measured in a Representative Patient Population. Chetlen AL, Brown KL, King SH, Kasales CJ, Schetter SE, Mack JA, Zhu J. AJR Am J Roentgenol. 2016 Feb;206(2):359-64; quiz 365. doi: 10.2214/AJR.15.14921. PMID: 26797364

15. Breast cancer: 4 big questions. Woolston C. Nature. 2015 Nov 19;527(7578):S120. doi: 10.1038/527S120a. PMID: 26580163

16. A single-institution study of 117 pregnancy-associated breast cancers (PABC): Presentation, imaging, clinicopathological data and outcome. Langer A, Mohallem M, Stevens D, Rouzier R, Lerebours F, Chérel P. Diagn Interv Imaging. 2014 Apr;95(4):435-41. doi: 10.1016/j.diii.2013.12.021. Epub 2014 Jan 31. PMID: 24485752

17. Breast imaging of the pregnant and lactating patient: imaging modalities and pregnancy-associated breast cancer. Vashi R, Hooley R, Butler R, Geisel J, Philpotts L. AJR Am J Roentgenol. 2013 Feb;200(2):321-8. doi: 10.2214/ajr.12.9814. PMID: 23345353

18. Multidisciplinary approach to breast cancer diagnosed during pregnancy: maternal and neonatal outcomes. Córdoba O, Llurba E, Saura C, Rubio I, Ferrer Q, Cortés J, Xercavins J. Breast. 2013 Aug;22(4):515-9. doi: 10.1016/j.breast.2012.10.005. Epub 2012 Oct 30. PMID: 23116970

19. Reducing delay in the diagnosis of pregnancy-associated breast cancer: how imaging can help us. Taylor D, Lazberger J, Ives A, Wylie E, Saunders C. J Med Imaging Radiat Oncol. 2011 Feb;55(1):33-42. doi: 10.1111/j.1754-9485.2010.02227.x. PMID: 21382187

20. Accuracy of diagnostic mammography and breast ultrasound during pregnancy and lactation. Robbins J, Jeffries D, Roubidoux M, Helvie M. AJR Am J Roentgenol. 2011 Mar;196(3):716-22. doi: 10.2214/AJR.09.3662. PMID: 21343518

21. Increased risk of breast cancer in multiparous and lactating women attending a breast care clinic in Pakistan: a paradigm shift? Fatima N, Zaman MU, Fatima T. Asian Pac J Cancer Prev. 2010;11(5):1219-23. PMID: 21198266

22. Assessment of mammographic density before and after first full-term pregnancy. Loehberg CR, Heusinger K, Jud SM, Haeberle L, Hein A, Rauh C, Bani MR, Lux MP, Schrauder MG, Bayer CM, Helbig C, Grolik R, Adamietz B, Schulz-Wendtland R, Beckmann MW,

23. Pregnancy-associated breast cancer: spectrum of imaging appearances. Ayyappan AP, Kulkarni S, Crystal P. Br J Radiol. 2010 Jun;83(990):529-34. doi: 10.1259/bjr/17982822. Epub 2010 Mar 24. PMID: 20335428

24. Pathological complete response in triple negative poorly differentiated invasive ductal breast carcinoma detected during pregnancy. Agrawal G, Chen JH, Baick CH, Chen AE, Mehta RS, Nalcioglu O, Su MY. J Clin Oncol. 2007 Jun 20;25(18):2618-20. doi: 10.1200/JCO.2007.11.3084. PMID: 17577043 No abstract available.

25. Mammography and fetal dose. Behrman RH, Homer MJ, Yang WT, Whitman GJ. Radiology. 2007 May;243(2):605; author reply 605-6. doi: 10.1148/radiol.2432060791. PMID: 17456884

26. Pregnancy-associated breast disease: radiologic features and diagnostic dilemmas. Son EJ, Oh KK, Kim EK. Yonsei Med J. 2006 Feb 28;47(1):34-42. doi: 10.3349/ymj.2006.47.1.34. PMID: 16502483

27. Imaging of breast cancer diagnosed and treated with chemotherapy during pregnancy. Yang WT, Dryden MJ, Gwyn K, Whitman GJ, Theriault R. Radiology. 2006 Apr;239(1):52-60. doi: 10.1148/radiol.2391050083. Epub 2006 Feb 16. PMID: 16484353

28. Rationale for a diagnostic chain in gestational breast tumor diagnosis. Bock K, Hadji P, Ramaswamy A, Schmidt S, Duda VF. Arch Gynecol Obstet. 2006 Mar;273(6):337-45. doi: 10.1007/s00404-005-0090-2. Epub 2005 Nov 26. PMID: 16311748

29. Coexistence of pregnancy and cancer. Jacobs IA, Chang CK, Salti GI. Am Surg. 2004 Nov;70(11):1025-9. PMID: 15586520

30. Pregnancy- and lactation-associated breast cancer: mammographic and sonographic findings. Ahn BY, Kim HH, Moon WK, Pisano ED, Kim HS, Cha ES, Kim JS, Oh KK, Park SH. J Ultrasound Med. 2003 May;22(5):491-7; quiz 498-9. doi: 10.7863/jum.2003.22.5.491. PMID: 12751860

31. The lactating breast: MRI findings and literature review. Talele AC, Slanetz PJ, Edmister WB, Yeh ED, Kopans DB. Breast J. 2003 May-Jun;9(3):237-40. doi: 10.1046/j.1524-4741.2003.09322.x. PMID: 12752635

32. Mammographic appearance of the breasts during pregnancy and lactation: false assumptions. Swinford AE, Adler DD, Garver KA. Acad Radiol. 1998 Jul;5(7):467-72. doi: 10.1016/s1076-6332(98)80186-4. PMID: 9653462

33. Samuels TH, Liu FF, Yaffe M, Haider M. Gestational breast cancer. Can Assoc Radiol J 1998; 49:172-180.

34. Perinatal characteristics and adult mammographic patterns. Ekbom A, Thurfjell E, Hsieh CC, Trichopoulos D, Adami HO. Int J Cancer. 1995 Apr 10;61(2):177-80. doi: 10.1002/ijc.2910610206. PMID: 7705944

35. Imaging of pregnancy-associated breast cancer. Liberman L, Giess CS, Dershaw DD, Deutch BM, Petrek JA. Radiology. 1994 Apr;191(1):245-8. doi: 10.1148/radiology.191.1.8134581. PMID: 8134581

36. Clinicopathologic characteristics and prognosis of breast cancer patients associated with pregnancy and lactation: analysis of case-control study in Japan. Ishida T, Yokoe T, Kasumi F, Sakamoto G, Makita M, Tominaga T, Simozuma K, Enomoto K, Fujiwara K, Nanasawa T, et al. Jpn J Cancer Res. 1992 Nov;83(11):1143-9. doi: 10.1111/j.1349-7006.1992.tb02737.x. PMID: 1483929

37. US as the Primary Imaging Modality in the Evaluation of Palpable Breast Masses in Breastfeeding Women, Including Those of Advanced Maternal Age. Chung M, Hayward JH, Woodard GA, Knobel A, Greenwood HI, Ray KM, Joe BN, Lee AY. Radiology. 2020 Nov;297(2):316-324. doi: 10.1148/radiol.2020201036. Epub 2020 Sep 1. PMID: 32870133

38. MRI versus mammography for breast cancer screening in women with familial risk (FaMRIsc): a multicentre, randomised, controlled trial. Saadatmand S, Geuzinge HA, Rutgers EJT, Mann RM, de Roy van Zuidewijn DBW, Zonderland HM, Tollenaar RAEM, Lobbes MBI, Ausems MGEM, van 't Riet M, Hooning MJ, Mares-Engelberts I, Luiten EJT, Heijnsdijk EAM, Verhoef C, Karssemeijer N, Oosterwijk JC, Obdeijn IM, de Koning HJ, Tilanus-Linthorst MMA; FaMRIsc study group. Lancet Oncol. 2019 Aug;20(8):1136-1147. doi: 10.1016/S1470-2045(19)30275-X. Epub 2019 Jun 17. PMID: 31221620

39. Palpable masses in breast during lactation. Obenauer S, Dammert S. Clin Imaging. 2007 Jan-Feb;31(1):1-5. doi: 10.1016/j.clinimag.2006.10.005. PMID: 17189838

40. Calcifications associated with lactational changes of the breast: mammographic findings with histologic correlation. Mercado CL, Koenigsberg TC, Hamele-Bena D, Smith SJ. AJR Am J Roentgenol. 2002 Sep;179(3):685-9. doi: 10.2214/ajr.179.3.1790685. PMID: 12185045

Appendix 2. Reasons for exclusion of retrieved studies

**Table 3:** Reasons for exclusion of retrieved studies

	Study	Reason of exclusion
1	Peterson et al. 2023	Case study
2	Zheng et al. 2023	Study content
3	Schad et al. 2020	Case report
4	Taron et al. 2019	Study content
5	Saadatmand et al. 2019	Study content
6	Khalil et al. 2018	Case report
7	Knabben et al. 2017	Review article
8	Chetlen et al. 2016	Study content
9	Woolston 2015	Study content
10	Vashi et al. 2013	Review article
11	Fatima et al. 2010	Study content
12	Loehberg et al. 2010	Study content
13	Ayyappan et al. 2010	Review article
14	Agrawal et al. 2007	Case report
15	Behrman et al. 2007	Study content
16	Jacobs et al. 2004	Study content
17	Talele et al. 2003	Case Report
18	Mercado et al. 2002	Case Report
19	Swinford et al. 1998	Study content
20	Ekbom et al. 1995	Study content

Appendix 3: Risk of bias quality assessment of the 20 included studies according to the ROBINS-1 tool pre-intervention and at-intervention domains

**Table 4:** Risk of bias quality assessment of the 20 included studies according to the ROBINS-1 tool pre-intervention and at-intervention domains SR = Serious risk, CR = Critical risk, MR = Moderate Risk, L = Low Risk, 1 = Confounding inherently not controllable, 2 = Selection into the study was very strongly related to intervention and outcome, 3 = (ii) Major aspects of the assignments of intervention status were determined in a way that could have been affected by knowledge of the outcome, 4 = Selection into the study was related (but not very strongly) to intervention and outcome and this could not be adjusted for in analyses, 5 = (i) Intervention status is well defined; and (ii) Intervention definition is based solely on information collected at the time of intervention.

Study	Domain 1 Bias due to confounding	Domain 2 Bias in selection of participants	Domain 3 Bias in classification of interventions
Hu et al. 2021	CR^ 1^	CR ^2^	SR^ 3^
Chung et al. 2020	CR^ 1^	SR^ 4^	LR^ 5^
Reyes et al. 2020	CR^ 1^	CR ^2^	SR^ 3^
Taşkın et al. 2019	CR^ 1^	CR ^2^	SR^ 3^
Wang et al. 2019	CR^ 1^	CR ^2^	SR^ 3^
Johansson et al. 2019	CR^ 1^	CR ^2^	SR^ 3^
Pugh et al. 2018	CR^ 1^	CR ^2^	SR^ 3^
Myers et al. 2017	CR^ 1^	SR^ 4^	LR^ 5^
Langer et al. 2014	CR^ 1^	CR ^2^	SR^ 3^
Córdoba et al. 2013	CR^ 1^	CR ^2^	SR^ 3^
Taylor et al. 2011	CR^ 1^	CR ^2^	SR^ 3^
Robbins et al. 2011	CR^ 1^	SR^ 4^	LR^ 5^
Son et al. 2006	CR^ 1^	CR ^2^	SR^ 3^
Yang et al. 2006	CR^ 1^	CR ^2^	SR^ 3^
Bock et al. 2006	CR^ 1^	SR^ 4^	LR^ 5^
Obenauer & Dammert 2006	CR^ 1^	CR ^2^	SR^ 3^
Ahn et al. 2003	CR^ 1^	CR ^2^	SR^ 3^
Samuels et al. 1998	CR^ 1^	CR ^2^	SR^ 3^
Liberman et al. 1994	CR^ 1^	CR ^2^	SR^ 3^
Ishida et al. 1992	CR^ 1^	CR ^2^	SR^ 3^

Appendix 4: Risk of bias quality assessment of the 20 included studies according to the ROBINS-1 tool post-intervention domains

**Table 5:** Risk of bias quality assessment of the 20 included studies according to the ROBINS-1 tool post-intervention domains SR = Serious risk, CR = Critical risk, MR = Moderate Risk, L = Low Risk, 1 = (i) There were deviations from intended intervention, but their impact on the outcome is expected to be slight, 2 = (i) (Unusual) There were critical differences between interventions in participants with missing data; and (ii) Missing data were not, or could not, be addressed through appropriate analysis, 3 = The methods of outcome assessment were so different that they cannot reasonably be compared across intervention groups, 4 = (ii) There is a high risk of selective reporting from among multiple analyses, 5 = (i) Any deviations from intended intervention reflected usual practice, 6 = (ii) The analysis is unlikely to have removed the risk of bias arising from the missing data, 7 = (10y minimally related to intervention status, 8 = (i) The outcome measurements and analyses are consistent with an a priori plan; or are clearly defined and both internally and externally consistent; and (ii) There is no indication of selection of the reported analysis from among multiple analyses; and (iii) There is no indication of selection of the cohort or subgroups for analysis and reporting on the basis of the results, 9 = There were deviations from usual practice that were unbalanced between the intervention groups and likely to have affected the outcome, 10 = (i) The methods of outcome assessment were comparable across intervention groups; and (ii) The outcome measure is only minimally influenced by knowledge of the intervention received by study participants; and (iii) Any error in measuring the outcome is only minimally related to intervention status.

Study	Domain 4 Bias due to deviations from intended interventions	Domain 5 Bias due to missing data	Domain 6 Bias in measurement of outcomes	Domain 7 Bias in selection of the reported result
Hu et al. 2021	MR^1^	CR^2^	CR^3^	SR^4^
Chung et al. 2020	LR^5^	MR^6^	MR^7^	MR^8^
Reyes et al. 2020	SR^9^	MR^6^	MR^10^	SR^4^
Taşkın et al. 2019	MR^1^	MR^6^	MR^7^	SR^4^
Wang et al. 2019	SR^9^	MR^6^	MR^10^	SR^4^
Johansson et al. 2019	SR^9^	CR^2^	CR^3^	SR^4^
Pugh et al. 2018	SR^9^	CR^2^	CR^3^	SR^4^
Myers et al. 2017	LR^5^	MR^6^	MR^7^	MR^8^
Langer et al. 2014	SR^9^	MR^6^	MR^10^	SR^4^
Córdoba et al. 2013	SR^9^	MR^6^	MR^10^	SR^4^
Taylor et al. 2011	SR^9^	MR^6^	MR^10^	SR^4^
Robbins et al. 2011	LR^5^	MR^6^	MR^7^	MR^8^
Son et al. 2006	SR^9^	MR^6^	MR^10^	SR^4^
Yang et al. 2006	SR^9^	MR^6^	MR^10^	SR^4^
Bock et al. 2006	LR^5^	MR^6^	MR^7^	MR^8^
Obenauer & Dammert 2006	SR^9^	MR^6^	MR^10^	SR^4^
Ahn et al. 2003	SR^9^	MR^6^	MR^10^	SR^4^
Samuels et al. 1998	SR^9^	MR^6^	MR^10^	SR^4^
Liberman et al. 1994	SR^9^	MR^6^	MR^10^	SR^4^
Ishida et al. 1992	SR^9^	MR^6^	MR^10^	SR^4^
